# Operations in the Nose and Naso-Pharynx

**Published:** 1908-11-21

**Authors:** 


### Anæsthetics.

### OPERATIONS IN THE NOSE AND NASO-PHARYNX.

For safe and successful administration of the anæsthetic for an operation in the upper air-passages it is necessary not only to take into consideration—as in all operations—such circumstances as the age and general state of health, but also to have a clear understanding with the surgeon as to his intentions, and to foresee any special dangers and difficulties of which there is even a remote possibility.

The anæsthetist should in every case inquire exactly what the surgeon proposes to do, especially if he is not accustomed to work with him. In almost every operation in this region surgeons differ, often considerably, as regards the position preferred, the depth and length of anæsthesia desired, the use of touch and sight, and other details of their procedures, and the anæsthetist should as far as possible arrange accordingly. A misunderstanding at a critical moment may cause delay and annoyance, or even serious trouble.

In many cases the anæsthetist has to perform various duties besides his own special work. There is often no room for an assistant, as the field of operation is so limited, and engages the attention of both surgeon and anæsthetist. He must, therefore, be prepared to fix the gag, steady the head in proper position, and act to some extent as an assistant, whilst he is at the same time watching carefully the effect of the anæsthesia and the safety of the patient.

Operations in this region should not be attempted with the patient lying in bed. It is extremely awkward for both surgeon and anæsthetist, and even in a case which promises to be quite simple, some unexpected complication may arise which cannot be dealt with in this position. For operations not done in the sitting position the patient should, unless there is very strong objection, be anæsthetised on the table—a steady anæsthesia cannot be maintained whilst lifting and carrying is going on, and vomiting is likely to occur.

Besides a mouth-wedge, props, gags, and tongue forceps, sponges on handles should be provided, and instruments should be always at hand for performance of laryngotomy or tracheotomy. These may not be wanted in a case in which difficulties are anticipated; on the other hand, they may be urgently needed in one expected to be trivial. Although these operations cannot be done under aseptic conditions, the anæsthetist should pay strict attention to the cleanliness of his hands and apparatus.

The required depth of anæsthesia varies according to circumstances, but it should never be so great as to abolish coughing and swallowing if there is probability of blood, pus, etc., entering the larynx. Too light anæsthesia, however, interferes with the operation and prolongs it, besides allowing inconvenient and even dangerous movements and reflex disturbances.

For operations lasting only a few seconds nitrous oxide gas (with air or oxygen) answers well in most cases, although rigidity or jactitation may cause inconvenience (especially with children), and it should never be used when there is much obstruction to the breathing, as, for instance, by inflammatory swelling in the throat. Ethyl-chloride gives a longer and quieter anæsthesia, but is undoubtedly more dangerous; and there is more liability to subsequent restlessness, headache, and sickness.

Ether, even when given for a few minutes only, is apt to cause increased vascularity and secretion of mucus, which is generally very objectionable to the operator. If selected for any special reason, such as the need for a sitting position, it should be given by the "open" method or preceded by nitrous oxide or ethyl-chloride, with frequent removal of the face piece subsequently to avoid cyanosis. Accidents may occur if the galvano-cautery be used after ether or ethyl-chloride has been administered. A.C.E or C.E does not usually give rise to trouble from hyperæmia, but in robust and florid subjects it is advisable to give chloroform from the beginning, particularly if there be an irritable state of the air-passages from smoking, alcoholism, or other cause. Chloroform, or a mixture containing it, should not, unless in very exceptional circumstances and with the utmost caution, be given with the patient in any position but the recumbent one, and (contrary to a prevalent belief) it is not safe for weakly children. Having regard to the fatalities which have occurred during its use for the removal of tonsils and adenoids it cannot be recommended for this purpose.

Whatever anæsthetic be employed, the administrator must obviate respiratory obstruction when present during induction by hooking up the chin, pushing the lower jaw forwards, drawing out the tongue, or some such manœuvre.

For enlarged tonsils and adenoids nitrous oxide, nitrous oxide with oxygen, and ethyl chloride are much used, especially when the surgeon likes to have the patient in a sitting position, to operate rapidly, and to see exactly what he is doing. Considerable practice is necessary to judge the degree of anæsthesia advisable when using chloride of ethyl. The mask should as a rule be removed as soon as there is commencing stertor, muscular relaxation, and dilatation of the pupils, without abolition of corneal reflex. If too much—even a little too much—is given, there is danger, on account of the abolition of the palatal and laryngeal reflexes, that blood will accumulate and will not be expelled by coughing; on the other hand insufficient anæsthetisation results in struggling or rigidity, and consciousness of pain, before the operation is satisfactorily completed. The anæsthetist standing behind the patient should see that the latter is properly seated and secured, and that there is nothing tight round the neck or waist.

A Doyen's gag is adjusted before application of the mask, notice being taken of loose teeth or gaps, with care not to nip the lips nor push back the tongue; cotton wool is useful to prevent air getting under the gag and also to prevent pressure on the cheek. The breathing may be embarrassed if it is opened at first to the full extent possible, but on removal of the mask the anæsthetist should do this in order that a good sweep of the curette may be made (avoiding tags), and he must hold it in position, also pushing the tonsils inwards and upwards and keeping the head steady. Opportunity should be taken to bend the head and shoulders forward to allow blood to escape, and if there is cyanosis or more than momentary intermission of breathing the pharynx must be quickly cleared and the gag loosened.

If chloroform or a chloroform-ether mixture be given, the child should be lying with the head on one side close to the table edge with the opposite shoulder raised, or else at first with the head straight and slightly extended. If over-extended there is too much strain on the neck and too much congestion and consequent hæmorrhage. Hyper-extension does not prevent inhalation of blood, and there is more risk of the tongue falling back. A small mouth-prop generally ensures freer breathing and makes it easier to put in the gag—or the latter may be put in but only slightly opened. The depth of anæsthesia is better known by the relaxation of muscles, the character of the breathing, and the absence of response to pinching, than by the corneal reflex, which is very deceptive in children. It should on no account be such as to cause pallor or dilatation with sluggishness of the pupils, and there should be some palatal reflex, which may be tested by the operator's finger. The child should as soon as possible be turned into a semi-prone position to allow escape of blood, and the gag should be relaxed. The pharynx may be gently sponged, but rough swabbing causes bruising of the palate and also prolongs the bleeding. The patient should be kept on the side and carefully watched until complete recovery has occurred; distressing fatalities have resulted from neglect of this precaution.

For operations within the nose a gag should always be inserted (although not at first opened) to allow the insertion of the operator's finger into the naso-pharynx, or removal of blood if required. Plugs should be removed before beginning the anæsthetisation. For short operations nitrous oxide, ethyl chloride, and ether permit the sitting position. For longer ones anæsthesia is maintained (with the patient recumbent) by chloroform in a Junker's apparatus, connected with a mouth-tube or one of the special gags made for the purpose. The latter give more freedom to the anæsthetist's hand, but difficulty occurs if the openings become blocked by the tongue or by blood or mucus, whereas a tube can be at once shifted or cleaned.

When a local anæsthetic has been efficiently applied, a light but steady general anæsthesia only is usually needed (when required at all) for such operations as resection of the septum. If it cannot be kept deep enough by the mouth tube, as occasionally happens in vigorous or alcoholic subjects, advantage should be taken of moments when the operator is not engaged to re-apply the face-piece; but too great depth must, of course, be avoided, especially if any blood is running backwards. If there is likely to be much bleeding, the patient may be kept with the head well on one side and slightly lowered, if not objectionable to the operator—or gauze plugs may be inserted in the posterior nares—or a sponge secured by tapes in the naso-pharynx. The last, however, causes coughing and retching unless the anæsthesia be deep.

The administration of an anæsthetic for these operations is not a task to be lightly undertaken. Success depends not only on great care and attention to details but—to a greater extent than is often realised—on the possession of skill, which can only come by practical experience.

